# Knowledge and Attitudes of Nurses Toward Genetics and Genomics: A Scoping Review

**DOI:** 10.7759/cureus.91196

**Published:** 2025-08-28

**Authors:** Konstantinos Mintzaridis, Evangelos Dousis, Eleni Evangelou, Maria Polikandrioti, Ioannis Koutelekos

**Affiliations:** 1 Department of Nursing, University of West Attica, Athens, GRC

**Keywords:** attitudes, genetic disorders, genetics, genomics, knowledge, nurses, nursing

## Abstract

Over the past two decades, genetics and genomics have emerged as a pivotal force in medicine and, consequently, healthcare systems. As the largest group of healthcare providers, nurses are essential in integrating genetics/genomics into clinical practice. Nurses' knowledge and attitudes toward genetics and genomics significantly influence their ability to integrate genetic/genomic principles into clinical practice.

The aim of this review was to identify existing literature and map key findings on attitudes and knowledge of nurses regarding genetics and genomics.

A scoping review of the literature published between 2013 and 2023 was conducted, encompassing four databases. A total of 1761 records were screened, and 49 studies met the inclusion criteria. Descriptive statistics and narrative synthesis were applied to summarize the studies' characteristics and findings.

The majority of the eligible studies focus on nurses' knowledge, both general and domain-specific, in genetics and genomics, while fewer studies focus specifically on attitudes. Less than a quarter of the studies (22.45%) were interventional. Findings revealed that basic knowledge among nurses was consistently low, often accompanied by misconceptions and a limited understanding of genetics. Studies that incorporated educational interventions demonstrated an increase in knowledge and a positive shift in attitudes.

Findings indicate that structured education in interventional studies improves nurses' knowledge and attitudes. These strategies have the potential to advance genomic competency among nurses and ultimately improve patient outcomes. This scoping review demonstrated that the subject remains under-researched, pointing to a significant gap for future research.

## Introduction and background

Two decades after the complete sequencing of the human genome, there are still efforts to understand the role of genes in biological processes and the interaction between genes and the environment, in order to apply knowledge for disease treatment [1,2]. The terms "genetics" and "genomics" refer to different concepts [3]. Genetics is the study of genes and their roles in inheritance, focusing on the way traits or conditions are passed from one generation to another and the relative effects on human body functions [3,4]. Genomics examines a person's genes (genome) and their interactions with each other and the environment [3,4]. This field explores complex diseases such as heart disease, asthma, diabetes mellitus, and cancer, which are influenced by a combination of genetic and environmental factors rather than individual genes [3,4].

Genetics and genomics contribute to personal and public health by facilitating the identification, prevention, and management of diseases and reproductive decisions (i.e., risk assessment of hereditary diseases, identification of genetic disorders in all age groups, genetic screening for disease predisposition, personalized drug therapy, genetic evaluation for cancer risk management) [4-7]. The field of genetic research has witnessed an extraordinary rate of advancement and continues to generate vast amounts of new information [8]. These developments underscore the urgent need for healthcare professionals, including nurses, to embrace a genetic approach to patient care.

Despite the substantial progress in genetics and genomics, a growing disparity exists between the available knowledge and its implementation in daily clinical practice by healthcare professionals [9]. Many non-geneticist healthcare professionals feel unprepared to engage with genetic testing, risk assessments, and interpreting results due to the complexity of genetic terminology [10,11]. Though nurses represent the largest group of healthcare professionals (with an increase of 8.5% over the past year and an anticipated growth of more than 40% by 2031), they have adopted a slower genetic perspective on diseases, as they often perceive it as a novel and unfamiliar domain [12]. To bridge this gap and provide more comprehensive care for patients with hereditary and genetic disorders, nurses must acquire a solid foundation in genetic terminology, concepts, skills, and emerging technologies [13].

Attitudes are shaped by a complex interplay of personal experiences, prior knowledge, beliefs, values, emotions, and external influences such as peers, media, and cultural factors [14,15]. A nurse's attitude toward genetics and genomics plays a pivotal role in their willingness to integrate new genetic knowledge into clinical practice [16].

Positive attitudes toward genetics and genomics may facilitate the adoption of new knowledge and practices by nursing personnel in hospitals [17,18]. Nurses with adequate knowledge in genetics and genomics are better equipped to understand the benefits, limitations, and implications of genetic medicine for patient care [17,18]. Conversely, a lack of knowledge and confidence may lead to reluctance, resistance, or fear of utilizing genetic approaches. This review aimed to explore attitudes and knowledge of nurses regarding genetics and genomics.

## Review

Methodology

This scoping review was conducted from January 2023 to January 2024, following the Preferred Reporting Items for Systematic Reviews and Meta-Analyses extension for Scoping Reviews (PRISMA-ScR) guidelines [19]. It aimed to identify and map, in the available literature, studies on nurses' knowledge and attitudes toward genetics and genomics.

A thorough literature search was conducted using four databases (PubMed, Scopus, ProQuest, and Google Scholar). Keywords (nurses, knowledge, attitudes, genetics, genomics, genetic disorders) were combined with Boolean operators (AND/OR) for conducting the database search.

Inclusion criteria for eligible studies included the following: original, peer-reviewed research studies; studies reporting findings from original studies; studies focusing on nurses or nursing activities; studies published in English; and studies published since 2013. In contrast, exclusion criteria included the following: grey literature (letters to the editor and commentary articles, case studies, training programs, policy papers); literature reviews; articles reporting secondary or tertiary sources; studies in which nurses participate in limited numbers; studies focused on patient perceptions; studies not published in English; and studies published prior to 2013.

After removing duplicate titles, articles underwent an independent review of title and abstract. The remaining articles underwent independent full-text review. Any discrepancies during the review process were resolved by a third independent reviewer.

Narrative synthesis for the discussion and descriptive statistics (i.e., percentages) were employed to summarize the studies' characteristics and findings.

Results

**Figure 1:**
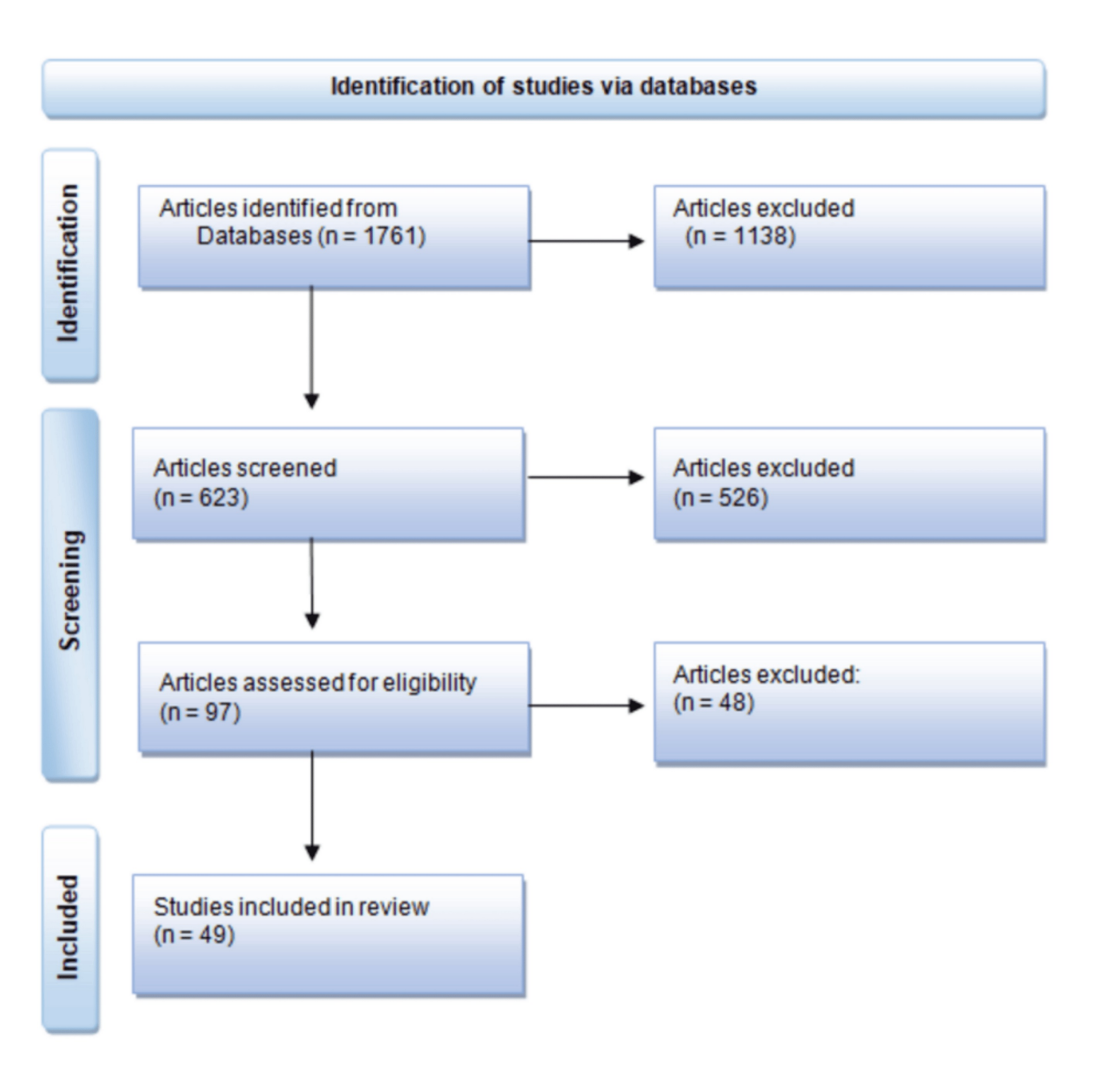
PRISMA flow diagram PRISMA: Preferred Reporting Items for Systematic Reviews and Meta-Analyses

A total of 1761 articles were identified. After excluding duplicates and articles not meeting the eligibility criteria, 623 articles remained. After screening titles and abstracts, 97 articles remained. From the 97 articles, only 49 were deemed, after full examination, to meet the inclusion criteria for this review (Figure 1).

**Figure 2:**
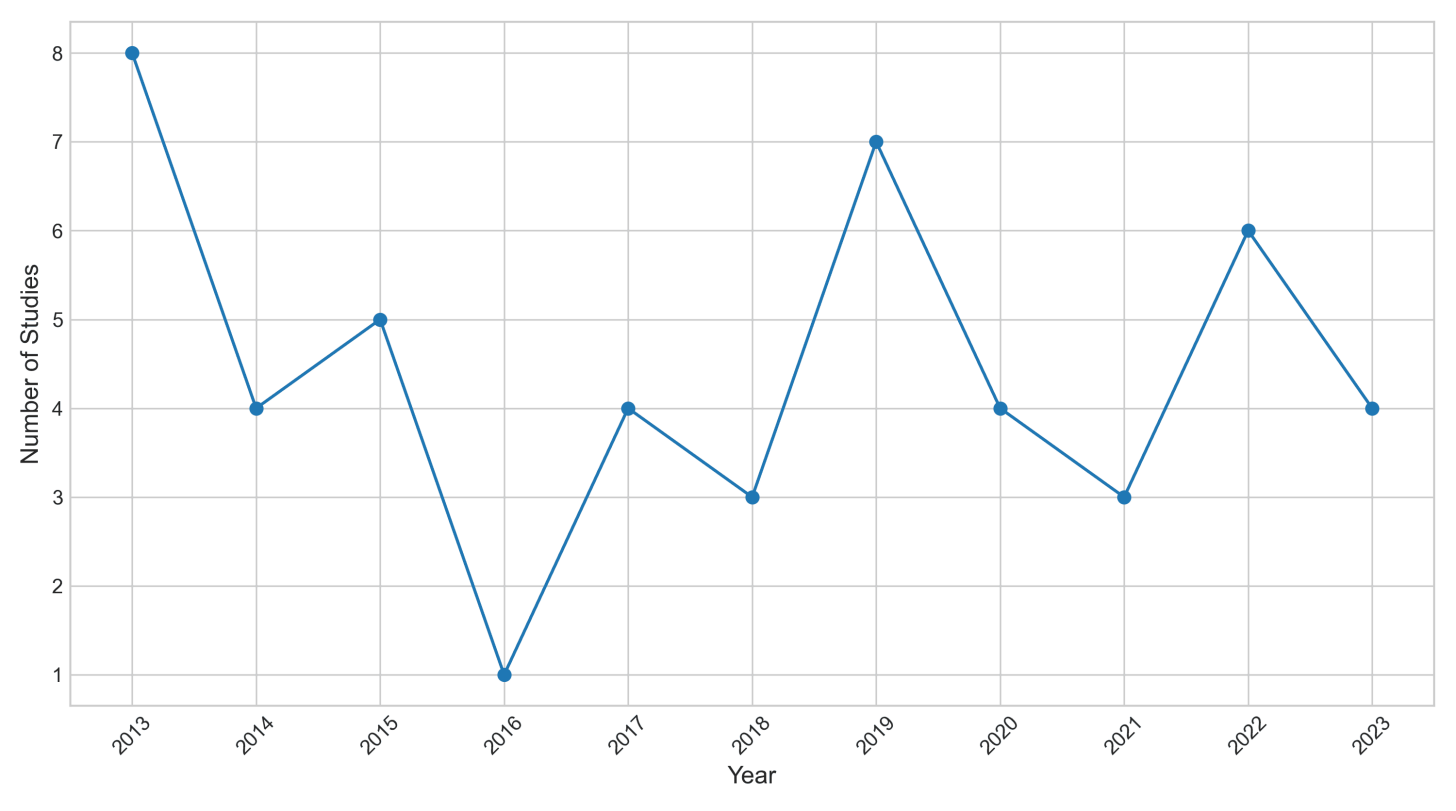
Annual distribution of studies

The distribution of studies included in this review spans from 2013 to 2023, reflecting a decade of research in the field of genetics and genomics. The distribution by year is as follows: 2013: eight studies (16.33%); 2014: four studies (8.16%); 2015: five studies (10.2%); 2016: one study (2.04%); 2017: four studies (8.16%); 2018: three studies (6.12%); 2019: seven studies (14.29%); 2020: four studies (8.16%); 2021: three studies (6.12%); 2022: six studies (12.24%); and 2023: four studies (8.16%). This distribution highlights the ongoing interest in genetic and genomic research over the years, with notable peaks in 2013 and 2019. The steady stream of studies each year underscores the sustained commitment to advancing knowledge and understanding in this critical field (Figure 2).

The studies included in this review focused on nurses, either exclusively or in combination with other health professionals [20-68]. Thirty-four out of 49 studies (69.39%) exclusively involved nurses [20,22-30,32,36-41,44,45,48,49,51-53,55,57-60,62-66], while 15 studies (30.61%) [21,31,33-35,42,43,46,47,50,54,56,61,67,68] involved a mixed population of nurses and other health professionals, specifically, nurses and physicians [43,46,50,61,67], nurses and oncologists [21], nurses, breast surgeons, and oncologists [31,68], nurses, physicians, and pharmacists [33,47,54], nurses and genetic counselors [34], nurses, medical geneticists, and obstetricians/gynecologists [35], nurses and midwives [42], and nurses, physicians, and dentists [56].

Regarding methodology, 37 studies (75.51%) [20,23-24,26-34,36,39,40,43,44,47-51,53-64,66-68] used a quantitative approach. A qualitative approach was used in six studies (12.24%) [25,35,38,42,46,52], and six studies used mixed methods (12.24%) [21,22,37,41,45,65].

**Figure 3:**
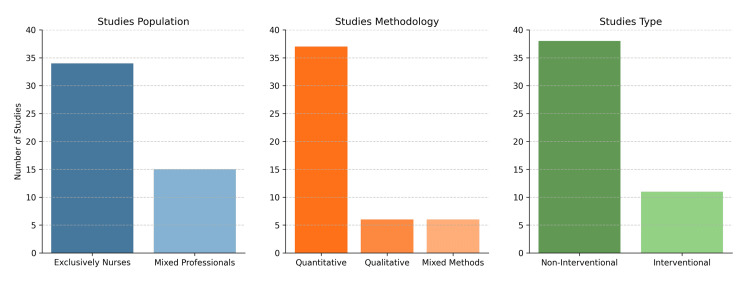
Summary of studies

The majority of studies were non-interventional (38/49, 77.55%) [20,22,24-26,28,29,32-35,38,39,42-44,46-50,52-55,56-68]. Eleven studies (22.45%) [21,23,27,30,31,36,37,40,41,45,51] were interventional, meaning they included an educational program aimed at the study population, with pre- and post-evaluation of knowledge and/or attitudes. This distinction between non-interventional and interventional studies highlighted the varied approaches in research, with a significant portion focusing on observational insights and a smaller, yet substantial, number implementing educational interventions to assess their impact on knowledge and attitudes in the field of genetics and genomics (Figure 3).

**Figure 4:**
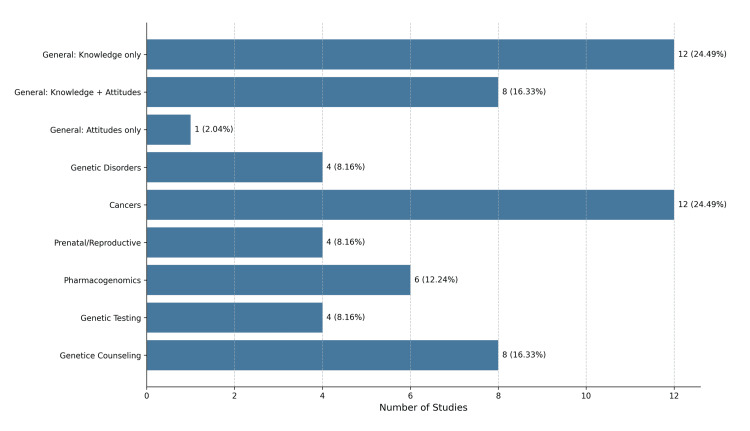
Distribution of studies by focus area (n=49)

The 49 studies included in this review were divided into two groups: Main Group 1 (knowledge and/or attitudes in general genetics/genomics) and Main Group 2 (knowledge and/or attitudes in specific domains of genetics/genomics). Specific areas of focus in Main Group 1 were general knowledge only, knowledge and attitudes, and attitudes only. Specific areas of focus in Main Group 2 were genetic disorders, cancers, prenatal care and reproductive endocrinology, pharmacogenetics/pharmacogenomics, genetic testing, and genetic counseling (Figure 4).

Main Group 1: Knowledge and/or Attitudes in General Genetics/Genomics

**Figure 5:**
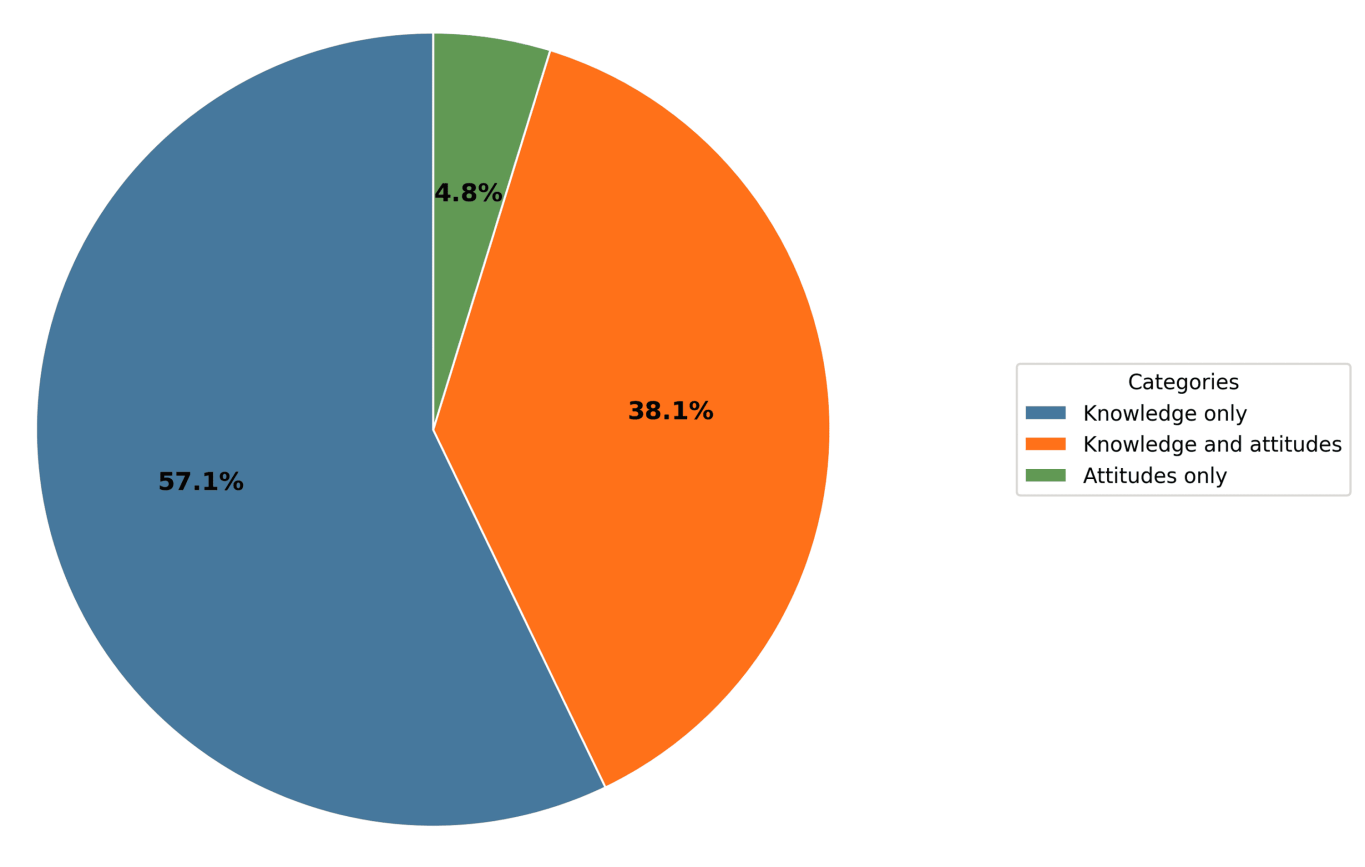
Distribution of studies in Main Group 1

The studies included in Main Group 1 evaluated nurses' overall knowledge, attitudes, or a combination of both, aiming to understand how well nurses grasp basic genetic concepts, inheritance patterns, and implications of genetic information. Assessment of attitudes was based on several factors, including negative and positive dispositions, confidence, comfort, and ethical considerations. Twelve studies [25,27,30,33,36,38,39,41,42,51,53,57] (57.14%) focused solely on knowledge. Eight studies [32,50,56,58,60,62,64,66] (38.10%) assessed both knowledge and attitudes, while only one study [63] (4.76%) concentrated exclusively on attitudes (Figure 5).

Main Group 2: Knowledge and/or Attitudes in Specific Domains of Genetics/Genomics

The studies included in Main Group 2 evaluated knowledge, attitudes, or a combination of both, in specific domains within genetics/genomics. Topics ranged from knowledge of disease-specific genetics, such as sickle cell disorder and hereditary cancers, to attitudes toward genetic counseling and prenatal testing. Main Group 2 includes the following sub-groups:

Genetic disorders: Knowledge and/or attitudes regarding specific genetic disorders were examined in four studies [40,43,44,67]. One study focused on nurses' knowledge about sickle cell disorder [40], and another on knowledge and attitudes toward managing sickle cell disorder patients [43]. Additionally, one study assessed nurses' knowledge about Berardinelli-Seip congenital lipodystrophy [44], while another examined attitudes toward genetic counseling for thalassemia major [67].

Cancers: Twelve of 49 studies (24.49%) examined knowledge and/or attitudes toward genetic aspects of cancer, including understanding genetic predispositions, hereditary cancer risks, the role of genetic mutations in cancer development, and the use of genetic testing for cancer risk assessment. Of these, seven studies focused on hereditary breast and ovarian cancer [21,22,31,48,55,61,68], while five studies examined hereditary cancer in general [24,28,34,46,59]. In terms of focus, knowledge was assessed toward breast cancer [48,55] and hereditary cancer more broadly [24,59], while combined knowledge and attitudes were evaluated in studies on breast cancer and ovarian cancer [22,31,48,61]. Several studies evaluated knowledge of genetic counseling in the context of cancer [21,34,46], whereas attitudes toward genetic testing for breast cancer were the main focus of one study [68]. Regarding participant populations, 11 of the included studies involved specialized nurses working in cancer care, while one study [24] examined both specialized and general practice nurses, and another [55] focused solely on general practice nurses.

Prenatal care and reproductive endocrinology: These studies explored knowledge and attitudes regarding prenatal care, which includes comprehensive monitoring and management of maternal and fetal health during pregnancy to ensure optimal outcomes for both. Four studies focused on different aspects of prenatal care. One study focused on attitudes toward prenatal genetic testing [35], while another analyzed knowledge surrounding genetics, ethics, and law [36]. A third study examined nurses' knowledge specifically related to prenatal care [49], and the fourth explored both the knowledge and attitudes of nurses in this context [37].

Pharmacogenetics/pharmacogenomics: These studies focused on knowledge and attitudes toward pharmacogenetics/pharmacogenomics, which includes understanding the relationship between genetics and drug response, how genetic variations can influence drug efficacy and safety, and attitudes about the use of pharmacogenetic testing in clinical practice. All six studies [20,29,45,47,52,54] explored nurses' knowledge in this field.

Genetic testing: These studies focused on knowledge and attitudes toward genetic testing, which involves the precise analysis of gene sequences to detect specific genetic mutations or variations that may predispose individuals to certain hereditary conditions or diseases. Four studies [35,37,63,68] focused on these aspects. Two of these studies explored attitudes toward treatment-focused genetic testing for women newly diagnosed with breast cancer [68], as well as attitudes toward noninvasive prenatal testing [35]. Additionally, one study [37] examined the level of knowledge on embryology and genetic testing, while another [63] investigated the attitudes of nurses toward genetic testing.

Genetic counseling: These studies focused on knowledge and attitudes toward genetic counseling services, exploring awareness of their benefits and processes. They also aimed to identify which healthcare professionals are responsible for providing counseling. Additionally, they assessed the perceived importance of these services in managing genetic conditions. Eight studies [21,23,26,30,31,34,65,67] explored this field. Five studies focused on the knowledge level regarding genetic counseling [21,23,30,31,65], while three examined attitudes toward genetic counseling [26,34,67] (Figure 4).

**Figure 6:**
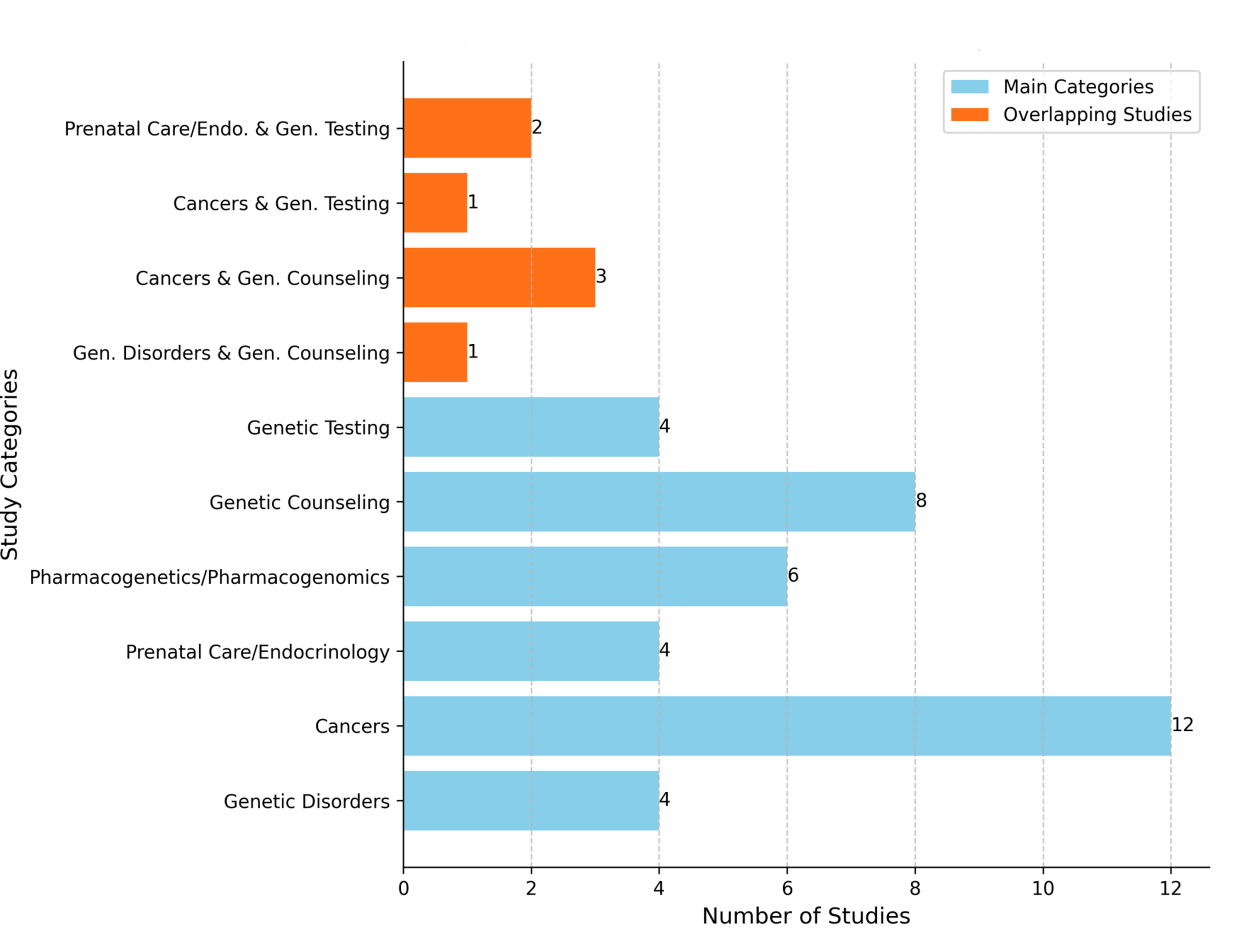
Distribution of studies and overlaps in Main Group 2

Considerable overlaps were evident across these fields. For example, cancer-related studies also examined attitudes toward genetic testing and counseling [68,21,31,34], studies of prenatal care often combined assessments of knowledge with evaluation of ethical and attitudinal factors [35,37], and studies on genetic counseling intersected with genetic testing [31,34,68] and genetic disorder [67] (Figure 6).

Discussion

This scoping review aimed to map relevant research on nurses' knowledge and attitudes toward genetics and genomics across diverse clinical aspects. The review provides a comprehensive overview of how nurses' knowledge and attitudes influence genomic competency in clinical practice.

At the dawn of the genomic era, nurses, as frontline health providers, play a crucial role in recognizing genetically related conditions, including single-gene inherited diseases such as sickle cell anemia, as well as genomic-related health issues like cancer, diabetes mellitus, and cardiovascular conditions [69,70]. Nurses are essential in obtaining comprehensive family histories, which help identify family members at risk for developing genetically influenced conditions and experiencing genomic-influenced drug reactions [69-71]. Their role also involves guiding individuals in making informed decisions about genetic and genomic tests and therapies, ensuring patients understand the results and implications of these tests [69-71]. Furthermore, nurses are responsible for referring at-risk individuals to appropriate healthcare professionals and agencies for specialized care [69,70].

Knowledge of Genetics/Genomics

The majority of the studies revealed that nurses lack essential knowledge in genetics, including basic genetic concepts, inheritance patterns, genetic diseases, risk identification, prenatal screening, genetic testing, comprehensive family history collection, and genetic counseling [23,25,32,33,38-41,50,51,53,56-58,60,62,64]. Apart from the lack of knowledge, misconceptions and inaccurate understanding of genetics and genomics are common among nurses [25,38,39,50,51,53,57,60,64]. These findings are consistent with previous studies, highlighting a persistent gap in genetic literacy among nurses [71-73]. Additionally, the review demonstrated a lack of knowledge for specific genetically related disorders such as Berardinelli-Seip congenital lipodystrophy [44], where most nurses had limited knowledge about the disorder. For hereditary cancers, nurses had low [22,28,59,61] or moderate knowledge [24,48,55], although the majority of the studies' population was nurses in oncologist departments. Furthermore, levels of knowledge about hereditary cancer were examined among cancer hospital nurses and general hospital nurses [24]. The overall genomic competency of cancer hospital nurses was comparable to that of general hospital nurses [24]. The majority of studies indicated that although nurses acknowledge the importance of genetics in hereditary cancers, a notable deficiency exists in their understanding of specific genetic principles, for example, the understanding that BRCA1 mutations, which are associated with breast and ovarian cancer in women, can also be inherited from the father [48]. This deficiency in knowledge and understanding of established genetic terms and findings poses a significant challenge for nurses [74,75]. It hinders their ability to integrate current genetic knowledge into practice, risking their relevance in a rapidly evolving genomics era [74,75]. As genetic advancements progress, staying updated on developments such as pharmacogenomics, whole genome sequencing (WGS), epigenetics, and proteomics will become increasingly challenging [74,75].

Knowledge of Pharmacogenetics/Pharmacogenomics

To further prove the truthfulness of the previous assessment, the studies in this review regarding the knowledge of nurses in pharmacogenetics/pharmacogenomics [20,29,45,47,52,54] indicated a poor understanding of pharmacogenomics among nurses. Most nurses had limited education in genetics and reported a poor understanding of pharmacogenetic testing in clinical practice [20,29,45,47,52,54]. Compared to other healthcare providers, nurses exhibited a larger deficit in knowledge [47,54]. For example, many nurses were unaware that genetic variations can account for up to 95% of the variability in drug disposition and effects [47]. The majority of nurses reported not ordering pharmacogenomic tests, not knowing which tests to order, and not using guidelines according to the Clinical Pharmacogenetics Implementation Consortium (CPIC) [20]. Additionally, there was a lack of confidence in interpreting pharmacogenomic test results and applying these findings to clinical care [20,45,47,52,54]. It remains unclear whether nurses perceive pharmacogenomics as important to nursing practice.

Attitudes Toward Genetics/Genomics

Most nurses expressed positive views about the impact of genetics/genomics in healthcare [25,32,53,58,60,62,64,66]. Yet they reported a lack of confidence in applying genetics/genomics, an indication of limited knowledge in this area, despite the fact that they frequently engage in genetically related tasks, such as gathering family history, conducting risk assessments, or interpreting genetic test results [38,42,56,58,62,64]. Additionally, concerns about the ethical and legal aspects of genetic information appear to critically influence nurses' attitudes to integrate genetics into practice [35,36,67].

Religious, moral, cultural, and historical beliefs significantly influence attitudes toward and access to specific genetic services, such as genetic testing [76]. Powell-Young and Spruill's study among members of the National Black Nurses Association revealed ambiguous attitudes toward genetics, with concerns about the potential for discriminatory use of genetic information, yet an acknowledgment of the importance of racial and ethnic minority participation in genetic research and testing [63]. Concerns about the potential discriminatory use of genetic information obtained through research and testing are frequently encountered among minority groups [77,78], largely due to a historical context of unethical eugenics practices [79] and the ongoing inadequate representation of these groups in genetic and genomic research [63,78]. The legacy of past abuses and the lack of inclusive research contribute to mistrust toward genetic studies within these communities [63,77,78]. Two studies [35,67] in this review also highlighted that religious beliefs are particularly intertwined with reproductive choices stemming from genetic information obtained through noninvasive prenatal testing [35,67]. Prenatal testing often presents the option of termination of pregnancy for fetal abnormalities, which faces strong religious opposition in many regions [35,67]. Furthermore, laws and regulations regarding abortion vary widely across different countries, for example, in Lebanon and Malaysia, abortion is illegal with exceptions, thus creating further barriers to addressing affected pregnancies [35,67,80-83].

Knowledge and Attitudes Toward Genetic Counseling

Attitudes toward genetic counseling were, in general, positive, emphasizing on the potential positive effect of genetic health information [21,23,26,30,31,34,41,67]. There were significant knowledge gaps and misconceptions regarding genetic counseling [30,31,65,67]. The majority of nurses believed that genetic counseling was only an informative and advisory process and could not specify the recipient of counseling [21,30,31,65]. Also, regarding the role of nurses in genetic counseling, the majority believed that they had no role, and only a few believed that nurses could provide information, support, and counseling [26,34,65]. Moreover, results suggest a lack of communication skills and confidence to effectively announce test results to patients or support them in making decisions [65,67]. Nurses have a tendency to refer patients to genetic services rather than counseling themselves [65]. Concerns about the potential of genetic information to trigger psychological burden on patients were frequently reported, despite the fact that genetic counseling reduces anxiety and improves the accuracy of perceived genetic risk [84].

Impact of Interventional Studies

A key finding of this review is that educational programs significantly improve nurses' perceived knowledge and confidence in applying genetics/genomics. Overall, the results indicate that nurses benefit substantially from exposure to genetics-/genomics-focused educational interventions [21,23,27,30,31,36,37,40,41,45,51].

Strengths and Limitations of the Study

This review provides a comprehensive synthesis of nurses' knowledge and attitudes toward genetics, highlighting key gaps and implications for practice and research. This review offers a broad overview rather than an in-depth analysis. It does not assess the risk of bias due to the methodological variability of the included studies. Additionally, an extensive search of the grey literature was not conducted. While this review primarily focused on nurses, some articles covering other professionals in addition to nurses were included to ensure that important information was not excluded.

## Conclusions

Evidences from this review indicate that while nurses recognize the significance of genetics and genomics in healthcare, they often lack essential knowledge, with misconceptions and inaccuracies being common. Positive attitudes toward genetics and genomics are tempered by these knowledge gaps as well as by ethical, cultural, and historical considerations. Interventional studies, though comprising less than a quarter of the reviewed literature, consistently demonstrated notable improvements in nurses' genomic knowledge, confidence, and perceived competence. This underscores the need for further research to confirm and expand the effectiveness of educational interventions in enhancing genomic competency. It also highlights the importance of ongoing education and support to enable nurses to integrate genetics into clinical practice effectively and advance toward a genomic era in healthcare.

## References

[1] Lander ES, Linton LM, Birren B (2001). Initial sequencing and analysis of the human genome. Nature.

[2] Claussnitzer M, Cho JH, Collins R (2020). A brief history of human disease genetics. Nature.

[3] (2024). Genetics vs. genomics fact sheet. https://www.genome.gov/about-genomics/fact-sheets/Genetics-vs-Genomics.

[4] Burton H, Jackson C, Abubakar I (2014). The impact of genomics on public health practice. Br Med Bull.

[5] Haga SB, Kim E, Myers RA, Ginsburg GS (2019). Primary care physicians' knowledge, attitudes, and experience with personal genetic testing. J Pers Med.

[6] Tsimberidou AM, Fountzilas E, Nikanjam M, Kurzrock R (2020). Review of precision cancer medicine: evolution of the treatment paradigm. Cancer Treat Rev.

[7] (2023). Fast Facts on Genetics and Genomics for Nurses: Practical Applications. https://catalog.nlm.nih.gov/discovery/fulldisplay?docid=alma9918401938306676&context=L&vid=01NLM_INST:01NLM_INST&lang=en&adaptor=Local%20Search%20Engine&tab=LibraryCatalog&query=lds56,contains,Genetic%20Testing%20--%20methods,AND&mode=advanced&offset=20.

[8] Hardiman G (2020). An introduction to systems analytics and integration of big omics data. Genes (Basel).

[9] Schaibley VM, Ramos IN, Woosley RL, Curry S, Hays S, Ramos KS (2022). Limited genomics training among physicians remains a barrier to genomics-based implementation of precision medicine. Front Med (Lausanne).

[10] Scheuner MT, Sales P, Hoggatt K, Zhang N, Whooley MA, Kelley MJ (2023). Genetics professionals are key to the integration of genetic testing within the practice of frontline clinicians. Genet Med.

[11] Mikat-Stevens NA, Larson IA, Tarini BA (2015). Primary-care providers' perceived barriers to integration of genetics services: a systematic review of the literature. Genet Med.

[12] Walker T, Ersig AL, Dwyer AA, Kronk R, Snyder CT, Whitt K, Willis V (2024). Integrating genomics and precision health knowledge into practice: a guide for nurse practitioners. J Am Assoc Nurse Pract.

[13] Kawasaki H, Kawasaki M, Iki T, Matsuyama R (2021). Genetics education program to help public health nurses improve their knowledge and enhance communities' genetic literacy: a pilot study. BMC Nurs.

[14] Ajzen I, Fishbein M (2011). Attitudes and the attitude-behavior relation: reasoned and automatic processes. Eur Rev Soc Psychol.

[15] Reid N, Amanat Ali A (2020). Beliefs and attitudes: why do attitudes matter?. Making Sense of Learning: A Research-Based Approach.

[16] White S, Jacobs C, Phillips J (2020). Mainstreaming genetics and genomics: a systematic review of the barriers and facilitators for nurses and physicians in secondary and tertiary care. Genet Med.

[17] Jaeger J, Hellwig A, Schiavoni E (2022). Challenges in improving genomic literacy: results from national and regional surveys of genomic knowledge, attitudes, concerns, and behaviors [PREPRINT]. bioRxiv.

[18] Khaerunnissa I, Setiawan H (2022). The influence of knowledge and work attitude on work motivation and its impact on the performance of nurses at RSUD Dr. Adjidarmo Rangkasbitung, Lebak District. Transforma Jurnal Manajemen.

[19] Tricco AC, Lillie E, Zarin W (2018). PRISMA Extension for Scoping Reviews (PRISMA-ScR): checklist and explanation. Ann Intern Med.

[20] Fulton CR, Macagno AL, Dickinson SL, Calzone K (2024). Advanced practice nurse pharmacogenomics capacity and utilization. J Am Assoc Nurse Pract.

[21] Bokkers K, Bleiker EM, Aalfs CM (2023). Surgical oncologists and nurses in breast cancer care are ready to provide pre-test genetic counseling. Ann Surg Oncol.

[22] Matsumoto M, Sasaki N, Tsukigawa Y, Otsubo R, Yano H, Nagayasu T (2023). A survey of the awareness and educational needs of nurses in Nagasaki Prefecture regarding hereditary breast and ovarian cancer. J Cancer Educ.

[23] Ahmed Mohamed H, Ahmed Mohamed N, ELSayed Ghonaem S, Hamdy Hafez S (2023). Nursing educational guidelines to enhance competency-based practice among nurses as genetic counselors. Egypt J Health Care.

[24] Zhao X, Li X, Liu Y, Calzone K, Xu J, Xiao X, Wang H (2022). Genetic and genomic nursing competency among nurses in tertiary general hospitals and cancer hospitals in mainland China: a nationwide survey. BMJ Open.

[25] Mikkelsen TR, Breer CB, Nissen KK, Christiansen K (2022). Understanding genetics in nursing care - a qualitative interview study. J Nurs Educ Pract.

[26] Obayashi C, Asahara K, Umeda M (2022). Difficulties in providing genetic consultations by public health nurses in Japan. Public Health Nurs.

[27] Vandiver KM, Erdei E, Mayer AG, Ricciardi C, O'Leary M, Burke K, Zelikoff JT (2022). Building environmental health and genomics literacy among healthcare providers serving vulnerable communities: an innovative educational framework. Int J Environ Res Public Health.

[28] Hébert J, Bergeron AS, Veillette AM, Bouchard K, Nabi H, Dorval M (2022). Issues associated with a hereditary risk of cancer: knowledge, attitudes and practices of nurses in oncology settings. Can Oncol Nurs J.

[29] Swadas N, Dewell S, Davidson SJ (2022). Knowledge and attitudes of pharmacogenetics among Canadian nurses: implications for nursing education. Quality Advancement in Nursing Education - Avancées En Formation Infirmière.

[30] Quinonez SC, O'Connor BC, Jacobs MF (2021). The introduction of genetic counseling in Ethiopia: results of a training workshop and lessons learned. PLoS One.

[31] van der Giessen J, Fransen MP, Spreeuwenberg P, Velthuizen M, van Dulmen S, Ausems MG (2021). Communication about breast cancer genetic counseling with patients with limited health literacy or a migrant background: evaluation of a training program for healthcare professionals. J Community Genet.

[32] Dagan E, Amit Y, Sokolov L, Litvak P, Barnoy S (2021). Integrating genomic professional skills into nursing practice: results from a large cohort of Israeli nurses. J Nurs Scholarsh.

[33] Almomani BA, Al-Sawalha NA, Al-Keilani MS, Aman HA (2020). The difference in knowledge and concerns between healthcare professionals and patients about genetic-related issues: a questionnaire-based study. PLoS One.

[34] Hickey M, Rio I, Trainer A, Marino JL, Wrede CD, Peate M (2020). What information do healthcare professionals need to inform premenopausal women about risk-reducing salpingo-oophorectomy?. Menopause.

[35] Haidar H, Vanstone M, Laberge AM, Bibeau G, Ghulmiyyah L, Ravitsky V (2020). Implementation challenges for an ethical introduction of noninvasive prenatal testing: a qualitative study of healthcare professionals' views from Lebanon and Quebec. BMC Med Ethics.

[36] Shin G, Jun M, Kim HK, Wreen M, Kubsch SM (2020). Key competencies for Korean nurses in prenatal genetic nursing: experiential genetic nursing knowledge, and ethics and law. J Educ Eval Health Prof.

[37] Catherino AB, Halupa C, Sharara FI, Bromer JG, Hayward B, Catherino WH (2019). Evaluation of an embryology and genetic testing patient counseling education intervention for reproductive endocrinology nurses. Fertil Steril.

[38] Goda H, Kawasaki H, Masuoka Y, Kohama N, Rahman MM (2019). Opportunities and challenges of integrating genetics education about human diversity into public health nurses' responsibilities in Japan. BMC Nurs.

[39] Wright H, Zhao L, Birks M, Mills J (2019). Genomic literacy of registered nurses and midwives in Australia: a cross‐sectional survey. J Nurs Scholarsh.

[40] Yacoub MI, Zaiton HI, Abdelghani FA, Elshatarat RA (2019). Effectiveness of an educational program on nurses' knowledge and practice in the management of acute painful crises in sickle cell disease. J Contin Educ Nurs.

[41] Jackson L, O'Connor A, Paneque M (2019). The Gen-Equip Project: evaluation and impact of genetics e-learning resources for primary care in six European languages. Genet Med.

[42] Saleh M, Kerr R, Dunlop K (2019). Scoping the scene: what do nurses, midwives, and allied health professionals need and want to know about genomics?. Front Genet.

[43] Balelah SH, Alawaji OM, Alhejaili NS (2019). Health care provider attitude during the management of sickle cell disease patients, a multicenter study in Saudi Arabia. Indo Am J Pharm Sci.

[44] Cândido Dantas VK, Soares JD, de Azevedo Medeiros LB (2018). Nurses' knowledge about Berardinelli-Seip congenital lipodystrophy. PLoS One.

[45] Dodson C (2018). Oncology nurses' knowledge of pharmacogenomics before and after implementation of an education module. Oncol Nurs Forum.

[46] Gonthier C, Pelletier S, Gagnon P (2018). Issues related to family history of cancer at the end of life: a palliative care providers' survey. Fam Cancer.

[47] Abdela OA, Bhagavathula AS, Gebreyohannes EA, Tegegn HG (2017). Ethiopian health care professionals' knowledge, attitude, and interests toward pharmacogenomics. Pharmgenomics Pers Med.

[48] Seven M, Pasalak SI, Guvenc G, Kok G (2017). Knowledge level and educational needs of Turkish oncology nurses regarding the genetics of hereditary breast and ovarian cancer. J Contin Educ Nurs.

[49] Seven M, Eroglu K, Akyüz A, Ingvoldstad C (2017). Educational needs of nurses to provide genetic services in prenatal care: a cross-sectional study from Turkey. Nurs Health Sci.

[50] Lopes-Júnior LC, Carvalho Júnior PM, de Faria Ferraz VE, Nascimento LC, Van Riper M, Flória-Santos M (2017). Genetic education, knowledge and experiences between nurses and physicians in primary care in Brazil: a cross-sectional study. Nurs Health Sci.

[51] Whitt KJ, Macri C, O'Brien TJ, Wright S (2016). Improving nurse practitioners' competence with genetics: effectiveness of an online course. J Am Assoc Nurse Pract.

[52] Dodson C (2015). Attitudes of oncology nurses concerning pharmacogenomics. Per Med.

[53] Seven M, Akyüz A, Elbüken B, Skirton H, Öztürk H (2015). Nurses' knowledge and educational needs regarding genetics. Nurse Educ Today.

[54] Kudzi W, Addy BS, Dzudzor B (2015). Knowledge of pharmacogenetics among healthcare professionals and faculty members of health training institutions in Ghana. Ghana Med J.

[55] Prolla CM, da Silva PS, Netto CB, Goldim JR, Ashton-Prolla P (2015). Knowledge about breast cancer and hereditary breast cancer among nurses in a public hospital. Rev Lat Am Enfermagem.

[56] Melo DG, de Paula PK, de Araujo Rodrigues S, da Silva de Avó LR, Germano CM, Demarzo MM (2015). Genetics in primary health care and the National Policy on Comprehensive Care for People with Rare Diseases in Brazil: opportunities and challenges for professional education. J Community Genet.

[57] Coleman B, Calzone KA, Jenkins J (2014). Multi-ethnic minority nurses' knowledge and practice of genetics and genomics. J Nurs Scholarsh.

[58] Saligan LN, Rivera RR (2014). Filipino-American nurses' knowledge, perceptions, beliefs and practice of genetics and genomics. Philipp J Nurs.

[59] Quinn GP, Knapp C, Sehovic I, Ung D, Bowman M, Gonzalez L, Vadaparampil ST (2014). Knowledge and educational needs about pre-implantation genetic diagnosis (PGD) among oncology nurses. J Clin Med.

[60] Calzone KA, Jenkins J, Culp S, Caskey S, Badzek L (2014). Introducing a new competency into nursing practice. J Nurs Regul.

[61] Pal T, Cragun D, Lewis C (2013). A statewide survey of practitioners to assess knowledge and clinical practices regarding hereditary breast and ovarian cancer. Genet Test Mol Biomarkers.

[62] Hsiao CY, Lee SH, Chen SJ, Lin SC (2013). Perceived knowledge and clinical comfort with genetics among Taiwanese nurses enrolled in a RN-to-BSN program. Nurse Educ Today.

[63] Powell-Young YM, Spruill IJ (2013). Views of Black nurses toward genetic research and testing. J Nurs Scholarsh.

[64] Calzone KA, Jenkins J, Culp S, Bonham VL Jr, Badzek L (2013). National nursing workforce survey of nursing attitudes, knowledge and practice in genomics. Per Med.

[65] Godino L, Turchetti D, Skirton H (2013). Genetic counseling: a survey to explore knowledge and attitudes of Italian nurses and midwives. Nurs Health Sci.

[66] Godino L, Turchetti D, Skirton H (2013). Knowledge of genetics and the role of the nurse in genetic health care: a survey of Italian nurses. J Adv Nurs.

[67] Ngim CF, Lai NM, Ibrahim H (2013). Counseling for prenatal diagnosis and termination of pregnancy due to thalassemia major: a survey of health care workers' practices in Malaysia. Prenat Diagn.

[68] Burcher S, Meiser B, Mitchell G (2013). Oncology health professionals' attitudes toward treatment-focused genetic testing for women newly diagnosed with breast cancer. Per Med.

[69] (2009). Essentials of Genetic and Genomic Nursing: Competencies, Curricula Guidelines, and Outcome Indicators. https://www.genome.gov/Pages/Careers/HealthProfessionalEducation/geneticscompetency.pdf.

[70] Calzone KA, Cashion A, Feetham S, Jenkins J, Prows CA, Williams JK, Wung SF (2010). Nurses transforming health care using genetics and genomics. Nurs Outlook.

[71] Terzioglu F, Dinç L (2004). Nurses' views on their role in genetics. J Obstet Gynecol Neonatal Nurs.

[72] Kim MY (2003). The nurses' knowledge and perception of their role in genetics. Taehan Kanho Hakhoe Chi.

[73] Calzone KA, Jenkins J, Bakos AD (2013). A blueprint for genomic nursing science. J Nurs Scholarsh.

[74] Alfaqih MA, Khader YS, Bashir N, Nusair Z, Nuseir Q, Nusier M (2019). Attitude of Jordanian physicians toward biochemistry and genetics. Biomed Res Int.

[75] Izzah SN, Setyanto D, Hasanatuludhhiyah N, Indiastuti DN, Nasution Z, d'Arqom A (2021). Attitudes of Indonesian medical doctors and medical students toward genome editing. J Multidiscip Healthc.

[76] Lea DH (2008). Genetic and genomic healthcare: ethical issues of importance to nurses. Online J Issues Nurs.

[77] Saulsberry K, Terry SF (2013). The need to build trust: a perspective on disparities in genetic testing. Genet Test Mol Biomarkers.

[78] Harris BH, McCabe C, Shafique H (2024). Diversity of thought: public perceptions of genetic testing across ethnic groups in the UK. J Hum Genet.

[79] Reilly PR (2015). Eugenics and involuntary sterilization: 1907-2015. Annu Rev Genomics Hum Genet.

[80] Gilani AI, Jadoon AS, Qaiser R (2007). Attitudes towards genetic diagnosis in Pakistan: a survey of medical and legal communities and parents of thalassemic children. Community Genet.

[81] Simpson B, Dissanayake VH, Wickramasinghe D, Jayasekara RW (2003). Prenatal testing and pregnancy termination in Sri Lanka: views of medical students and doctors. Ceylon Med J.

[82] Zahed L, Nabulsi M, Tamim H (2002). Attitudes towards prenatal diagnosis and termination of pregnancy among health professionals in Lebanon. Prenat Diagn.

[83] de Silva DC, Jayawardana P, Hapangama A (2008). Attitudes toward prenatal diagnosis and termination of pregnancy for genetic disorders among healthcare workers in a selected setting in Sri Lanka. Prenat Diagn.

[84] Meiser B, Halliday JL (2002). What is the impact of genetic counselling in women at increased risk of developing hereditary breast cancer? A meta-analytic review. Soc Sci Med.

